# Fungal infection strategies

**DOI:** 10.1080/21505594.2019.1682248

**Published:** 2019-11-14

**Authors:** Ilse D. Jacobsen

**Affiliations:** a Research Group Microbial Immunology, Leibniz Institute for Natural Product Research and Infection Biology, Hans Knöll Institute, Jena, Germany; b Institute of Microbiology, Friedrich Schiller University, Jena, Germany

Fungi are increasingly recognized as the cause of not only superficial infections, which affect many people but are relatively easy to treat, but also invasive and disseminated infections. Difficulties in diagnosing fungal infections and the limited number of antifungal drugs available contribute to the high mortality rates observed in these infections. Furthermore, severe fungal infections mainly occur in immunosuppressed individuals, for example, patients undergoing solid organ or stem cell transplantations, a group that has been increasing with the advancements of modern medicine resulting in increased numbers of severe fungal infections [1]. Immunosuppression as a risk factor highlights the important role of the immune system in controlling opportunistic fungal pathogens; it also suggests supporting the host immune functions or targeting interactions between the host immune system and fungi as alternative therapeutic strategies that could be combined with antifungal treatment [2]. For such approaches, however, a thorough understanding of host–pathogen interactions is necessary.

Human fungal pathogens are phylogenetically very diverse and belong to different phyla within the kingdom. The majority of severe infections, for example, are caused by members of the genera *Candida* and *Aspergillus*, belonging to the Ascomycota, and *Cryptococcus*, a basidiomycete. More rare, but very severe, are infections caused by various species belonging to the phylum Mucoromycotina. This suggests that pathogenicity evolved independently across the fungal kingdom several times and raises two main questions: (i) in which context did pathogenicity evolve, and (ii) are there common and specific virulence traits in phylogenetically diverse fungal pathogens [3].

A common feature of dermatophytes is the ability to utilize keratin as a nutrient source. Keratins as structural proteins of hair, nails, and epidermal layers of the skin are found not only on animal and human hosts but also in the environment, as keratinous structures are constantly shed as part of the bodies’ renewal process. Dermatophyte species are classified as geophilic, zoophilic and anthropophilic, depending on their preferred niche; interestingly, clinical infection tends to be more severe, driven by both fungus-driven host tissue destruction and overt inflammation, if dermatophytes colonize a human or animal theyare not adapted to [4]. An example is the occurrence of certain zoophilic dermatophytes, such as *Microsporium canis* and *Trichophytum benhamiae*, on asymptomatic animal hosts, while the same strains cause inflammatory reactions in humans [5]. This suggests that specific adaptation of these fungi to natural host species is resulting in commensal behaviour while in untypical hosts the immune response and possibly altered fungal behaviour are driving inflammation resulting in clinical disease. Although dermatophyte infections are the most common superficial fungal infection in animals and humans, genetic features of dermatophytes and factors that drive pathogenesis are, however, not well understood yet, and the mechanisms underlying host adaptation remain to be elucidated.

One explanation why deeply invasive or disseminated infections by dermatophytes are very rare is their limited tolerance to body core temperature, usually around 37.5°C in mammals. In contrast, other saprophytic environmental fungi, such as some *Aspergillus* species and several mucormycetes, are temperature-tolerant. This is likely the result of adaptation of the elevated temperatures found in decaying organic matter, such as compost heaps, which also represents the natural habitat of these fungi. Many *Aspergillus* and mucormycete species are found ubiquitously in the environment and have a versatile metabolism that allows them to thrive on various types of organic material. This Special Focus issue includes the paper of Lackner *et al*.[6] who investigated the correlation of temperature tolerance, resistance to oxidative stress, and growth rates of cryptic *Aspergillus* species of section Terrei for virulence in a wax moth larvae model. Using 73 isolates of six clinically relevant species they show that different strains of all species were well adapted to growth in this insect host and that virulence mainly correlated with growth rates at 37°C. Their data furthermore suggest that in this group of fungi virulence is a strain – rather than a species-specific feature.

In the environment, fungi however not only have to compete with other fungi, bacteria, and protozoa for nutrients but are also facing predators such as nematodes and amoeba. In their ability to take up and digest microorganisms, amoebas resemble mammalian phagocytes, and some of the mechanisms mediating prey recognition and killing by amoeba are also found in phagocytes. It thus appears likely that counterstrategies evolved in fungi in response to amoeba also enable them to prevent recognition or killing by mammalian phagocytes, and thus promote virulence, a concept also called “amoeboid predator-fungal animal virulence hypothesis” [7].

Not surprisingly, the fungal cell surface plays a key role in recognition by amoeba and phagocytes alike; on *Aspergillus fumigatus* spores, specific proteins (rodlets) and pigmentation convey protection from environmental stresses such as UV, but also hamper recognition by phagocytes [8,^9^]. Germination and growth of vegetative hyphae, however, results in the production or exposure of cell wall components such as galactosaminogalactan (GAG) and β-1,3-glucan [10]. In this special focus issue, Speth et al. review the impact of GAG on *Aspergillus* pathogenesis [11]. GAG is a heteropolysaccharide secreted by actively growing hyphae and binding to the surface of these hyphae. It mediates interaction with host factors, such as surfactant D, shields the underlying β-1,3 glucan from recognition by immune cells, interferes with neutrophil activity against *A. fumigatus*, and modulates cytokine and cell responses. Overall, GAG mediates immunosuppressive effects, which has led to an interest in the use of soluble GAG in the treatment of inflammatory diseases [11]. In contrast to GAG, β-1,3 glucan is a well-known ligand of Dectin-1, a C-type lectin pattern recognition receptor (PRR) known to be involved in antifungal immunity [12]. While the role of PRRs in antifungal defense has been extensively studied, the role of opsonization by complement is less well understood. This topic has been addressed by Steger *et al*. in work included in this Special Focus issue [13]. They demonstrate that complement deposition enhances recognition and phagocytosis of *A. fumigatus* conidia by dendritic cells. Not only melanin, the pigment contributing to immune evasion of *A. fumigatus* spores, interferes with complement deposition but also β-1,3 glucan, highlighting the complexity of interactions of the immune system with the fungal cell wall [13].

Pigmentation is also an important feature of *Cryptococcus neoformans* and *Cryptococcus gattii*, dimorphic basidiomycetes that grow as filaments in the environment but switch to yeast growth in warm-blooded hosts. Like *Aspergillus* and molds, *Cryptococcus* likely acquired its virulence traits in the environment as a mean to withstand adverse environmental conditions and predators [14]. It has not only become the fungal pathogen causing the highest number of severe human infections as a result of the HIV pandemic but is also known to infect a wide range of animals. In a review in this Special Focus issue, Oscar Zaragoza summarizes the principles of *Cryptococcus* virulence which, in addition to general adaptation to the host environment and melanin production, includes the production of a capsule and specific types of polysaccharides [15]. Another interesting feature of *Cryptococcus* is the formation of giant yeast cells, termed Titan cells that due to their size cannot be phagocytosed, and are more resistant to stressors [16]. Further examples presented in this Special Focus issue of opportunistic environmental fungal pathogens which produce pigments are *Exophiala dermatitidis*, reviewed by Kirchhoff *et al*.[17], and *Coccidioides. E. dermatitidis* is a black yeast, a term originating from the dark pigmentation of this group of fungi. It can cause phaeohyphomycosis in immunosuppressed patients, but also fatal infections in healthy patients. It is also regularly isolated from cystic fibrosis patients, but its role in disease in this patient group is unclear. Besides pigmentation, the secretion of extracellular polysaccharides (acid mucopolysaccharides), adherence and biofilm formation, and the ability to form invasive filaments contribute to virulence of this fungus [17]. Interestingly, *E. dermatitidis* is most commonly isolated from indoor man-made habitats, and although it is considered a ubiquitous fungus, isolation from the environment is rare. Its main environmental habitat is assumed to be tropical rainforests, but little is known about the natural habitat and the transmission routes. One can thus only speculate on the context in which the virulence traits of *E. dermatitidis* evolved, but it is likely the environmental niche, rather than an occasional association with humans. This is also the case for *Coccidioides immitis* and *C. posadasii*, the causative agents of coccidioidomycosis, a disease commonly known as valley fever [18].

Kollath *et al*. review our current understanding of both the disease and the pathogens’ biology which remains largely enigmatic to date [19]. *Coccidioides* infects immunocompetent mammals including humans, and although infection is commonly asymptomatic or results only in mild, self-limiting pneumonia, disseminated infections occur that require medical intervention. Like *Cryptococcus, Coccidioides* is dimorphic with filamentous growth in the environment while in the host spherules that produce endospores are formed. *C. immitis* and *C. posadasii* are endemic to areas with arid to semi-arid alkaline thermic soils throughout western North America and into Central and South America, and it appears that sufficiently high soil temperatures in combination with low water-holding capacity are required for these fungi. Although *Coccidioides* has been thought to be a saprotrophic soil-dwelling fungi, the exact conditions required for growth and maturation in the environment are unknown [19]. Recent evidence suggests that its natural life requires close association with small desert mammals such as rodents, and utilization of animal-derived substrates. *Coccidioides* was isolated from deer mice, pocket mice, ground squirrels, grasshopper mice, kangaroo rats, and pack rats; it thus appears possible that virulence in these fungi not only evolved in the environment as a consequence of encounters with predators like amoeba, but as a result of adaptation to rodents, with the close proximity facilitating infections [13].

From the examples described above, it appears that pigmentation is a common feature of environmental opportunistic fungal pathogens, mediating both protection in the environment and immune evasion in the host. Human pathogenic fungi of the genus *Candida*, however, do not produce any pigments. Furthermore, while some *Candida* species are found in the environment, such as *C. tropicalis* and *C. krusei*, others like *C. glabrata* and *C. albicans* have been isolated only or predominantly from skin or mucosal surfaces of warm-blooded animals, including humans. It thus appears that these fungi are evolutionarily adapted to a commensal lifestyle [20], albeit this is challenged by recent whole-genome analyses [21]. Adaptation to a host however clearly occurred in *Pneumocystis*, a group of fungi that are extremely difficult to grow outside a warm-blooded host and have evolved to host-specific species [22]. Humans are probably infected with *Pneumocystis* early in life, but clinical infections usually occur only if the host is severely immunocompromised [23].

Virulence of *C. albicans* is associated not only with metabolic adaptation and resistance to potential stressors in the host [24,25] but also the ability to switch between yeast and invasive filamentous growth forms associated with production of the peptide toxin Candidalysin [26,27]. The “classical” fitness properties and virulence factors of *Candida* have been reviewed extensively elsewhere [26,28,29]; in this Special Focus, we included a review by Chakraborty *et al*. on a topic less studied: Eicosanoid production by pathogenic yeasts [30]. Eicosanoids are bioactive lipid mediators generated in many mammalian cells from the oxidation of arachidonic acid and regulate various physiological functions, including cellular homoeostasis and modulation of immune responses. Various human pathogenic fungi are also able to produce eicosanoids, to some extent even the very same molecules as their mammalian hosts [30]. There is some evidence that fungi-derived lipid mediators affect the host immune response and thereby pathogenesis, albeit the exact function is yet to be unravelled. As eicosanoids are not only found in fungi associated with mammals but also environmental fungi and plant pathogens, it is likely that they did not primarily evolve as virulence traits but rather serve yet to be discovered functions in fungi themselves.

For *Candida* species occurring as commensals, it appears essential that virulence factors are regulated as damage associated with virulence factors might trigger the immune system and subsequent elimination of the fungus [31]. In plant pathogenic fungi factors have been identified that trigger a hypersensitive immune response in the infected plant, which promotes plant resistance and renders the pathogen avirulent [32]. Such factors have been termed “antivirulence factors” and the respective genes “avirulence genes” [32,33]. In the final paper of this Special Focus, Siskar-Lewin *et al*. discuss how this concept could be transferred to human pathogenic fungi and extend our understanding of host-fungal interaction [34].

## References

[1] Brown GD, Denning DW, Gow NA, et al. Hidden killers: human fungal infections. Sci Transl Med. 2012;4:165rv13. 10.1126/scitranslmed.3004404 23253612

[2] Wheeler ML, Limon JJ, Underhill DM. Immunity to commensal fungi: detente and disease. Annu Rev Pathol. 2017;12:359–385. 28068483 10.1146/annurev-pathol-052016-100342 PMC6573037

[3] Köhler JR, Hube B, Puccia R, et al. Fungi that infect humans. Microbiol Spectr. 2017;5:3. 10.1128/microbiolspec.funk-0014-2016 PMC11687496 28597822

[4] Hube B, Hay R, Brasch J, et al. Dermatomycoses and inflammation: the adaptive balance between growth, damage, and survival. J Mycol Med. 2015;25:e44–58. 25662199 10.1016/j.mycmed.2014.11.002

[5] Gräser Y, Monod M, Bouchara JP, et al. New insights in dermatophyte research. Med Mycol. 2018;56:2–9. 29538740 10.1093/mmy/myx141

[6] Lackner M, Obermair J, Naschberger V, et al. Cryptic species of *Aspergillus* section *Terrei* display essential physiological features to cause infection and are similar in their virulence potential in *Galleria mellonella*. Virulence. 2019;10:542–554. 31169442 10.1080/21505594.2019.1614382 PMC6592363

[7] Casadevall A, Fu MS, Guimaraes AJ, et al. The ‘Amoeboid predator-fungal animal virulence’ hypothesis. J Fungi (Basel). 2019;5:10. 10.3390/jof5010010 PMC6463022 30669554

[8] Aimanianda V, Bayry J, Bozza S, et al. Surface hydrophobin prevents immune recognition of airborne fungal spores. Nature. 2009;460:1117–1121. 19713928 10.1038/nature08264

[9] Hillmann F, Novohradska S, Mattern DJ, et al. Virulence determinants of the human pathogenic fungus *Aspergillus fumigatus* protect against soil amoeba predation. Environ Microbiol. 2015;17:2858–2869. 25684622 10.1111/1462-2920.12808

[10] Latge JP, Beauvais A, Chamilos G. The cell wall of the human fungal pathogen *Aspergillus fumigatus*: biosynthesis, organization, immune response, and virulence. Annu Rev Microbiol. 2017;71:99–116. 28701066 10.1146/annurev-micro-030117-020406

[11] Speth C, Rambach G, Lass-Flörl C, et al. Galactosaminogalactan (GAG) and its multiple roles in *Aspergillus* pathogenesis. Virulence. 2019;10:976–983. 10.1080/21505594.2019.1568174 PMC8647848 30667338

[12] Dambuza IM, Brown GD. C-type lectins in immunity: recent developments. Curr Opin Immunol. 2015;32:21–27. 25553393 10.1016/j.coi.2014.12.002 PMC4589735

[13] Steger M, Bermejo-Jambrina M, Yordanov T, et al. Beta-1,3-glucan-lacking *Aspergillus fumigatus* mediates an efficient antifungal immune response by activating complement and dendritic cells. Virulence. 2018;10:957–969. 10.1080/21505594.2018.1528843 PMC8647855 30372658

[14] Casadevall A, Steenbergen JN, Nosanchuk JD. ‘Ready made’ virulence and ‘dual use’ virulence factors in pathogenic environmental fungi–the *Cryptococcus neoformans* paradigm. Curr Opin Microbiol. 2003;6:332–337. 12941400 10.1016/s1369-5274(03)00082-1

[15] Zaragoza O. Basic principles of the virulence of *Cryptococcus*. Virulence. 2019;10:490–501. 31119976 10.1080/21505594.2019.1614383 PMC6550552

[16] Trevijano-Contador N, Rueda C, Zaragoza O. Fungal morphogenetic changes inside the mammalian host. Semin Cell Dev Biol. 2016;57:100–109. 27101887 10.1016/j.semcdb.2016.04.008

[17] Kirchhoff L, Olsowski M, Rath PM, et al. *Exophiala dermatitidis*: Key issues of an opportunistic fungal pathogen. Virulence. 2019;10:984–998. 10.1080/21505594.2019.1596504 PMC8647849 30887863

[18] Kirkland TN, Fierer J. *Coccidioides immitis* and *posadasii*; A review of their biology, genomics, pathogenesis, and host immunity. Virulence. 2018;9:1426–1435. 30179067 10.1080/21505594.2018.1509667 PMC6141143

[19] Kollath DR, Miller KJ, Barker BM. The mysterious desert dwellers: *coccidioides immitis* and *Coccidioides posadasii*, causative fungal agents of coccidioidomycosis. Virulence. 2019;10:222–233. 30898028 10.1080/21505594.2019.1589363 PMC6527015

[20] McManus BA, Coleman DC. Molecular epidemiology, phylogeny and evolution of *Candida albicans*. Infect Genet Evol. 2014;21:166–178. 24269341 10.1016/j.meegid.2013.11.008

[21] Gabaldon T, Fairhead C. Genomes shed light on the secret life of *Candida glabrata*: not so asexual, not so commensal. Curr Genet. 2019;65:93–98. 30027485 10.1007/s00294-018-0867-z PMC6342864

[22] Aliouat-Denis CM, Chabe M, Demanche C, et al. *Pneumocystis* species, co-evolution and pathogenic power. Infect Genet Evol. 2008;8:708–726. 18565802 10.1016/j.meegid.2008.05.001

[23] Gigliotti F, Limper AH, Wright T. Pneumocystis. Cold Spring Harb Perspect Med. 2014;4:a019828. 25367973 10.1101/cshperspect.a019828 PMC4292088

[24] Ene IV, Brunke S, Brown AJ, et al. Metabolism in fungal pathogenesis. Cold Spring Harb Perspect Med. 2014;4:a019695. 25190251 10.1101/cshperspect.a019695 PMC4292087

[25] Brown AJ, Budge S, Kaloriti D, et al. Stress adaptation in a pathogenic fungus. J Exp Biol. 2014;217:144–155. 24353214 10.1242/jeb.088930 PMC3867497

[26] Noble SM, Gianetti BA, Witchley JN. *Candida albicans* cell-type switching and functional plasticity in the mammalian host. Nat Rev Microbiol. 2017;15:96–108. 27867199 10.1038/nrmicro.2016.157 PMC5957277

[27] Jacobsen ID, Wilson D, Wachtler B, et al. *Candida albicans* dimorphism as a therapeutic target. Expert Rev Anti Infect Ther. 2012;10:85–93. 22149617 10.1586/eri.11.152

[28] Miramon P, Lorenz MC. A feast for *Candida*: metabolic plasticity confers an edge for virulence. PLoS Pathog. 2017;13:e1006144. 28182769 10.1371/journal.ppat.1006144 PMC5300112

[29] Sellam A, Whiteway M. Recent advances on *Candida albicans* biology and virulence. F1000Res. 2016;5:2582. 27853524 10.12688/f1000research.9617.1 PMC5089126

[30] Chakraborty T, Toth R, Gacser A. Eicosanoid production by *Candida parapsilosis* and other pathogenic yeasts. Virulence. 2018;10:970–975. 10.1080/21505594.2018.1559674 PMC8647850 30558484

[31] Witchley JN, Penumetcha P, Abon NV, et al. *Candida albicans* morphogenesis programs control the balance between gut commensalism and invasive infection. Cell Host Microbe. 2019;25:432–43.e6. 30870623 10.1016/j.chom.2019.02.008 PMC6581065

[32] Lo Presti L, Lanver D, Schweizer G, et al. Fungal effectors and plant susceptibility. Annu Rev Plant Biol. 2015;66:513–545. 25923844 10.1146/annurev-arplant-043014-114623

[33] Petit-Houdenot Y, Fudal I. Complex interactions between fungal avirulence genes and their corresponding plant resistance genes and consequences for disease resistance management. Front Plant Sci. 2017;8:1072. 28670324 10.3389/fpls.2017.01072 PMC5472840

[34] Siscar-Lewin S, Hube B, Brunke S. Antivirulence and avirulence genes in human pathogenic fungi. Virulence 2019;10:935–947. 10.1080/21505594.2019.1688753 PMC6930009 31711357

